# The exploration of mechanisms of comorbidity between migraine and depression

**DOI:** 10.1111/jcmm.14390

**Published:** 2019-05-20

**Authors:** Qing Zhang, Anwen Shao, Zhengyan Jiang, Huitzong Tsai, Weibo Liu

**Affiliations:** ^1^ Department of Psychiatry Second Affiliated Hospital, School of Medicine, Zhejiang University Hangzhou China; ^2^ Department of Neurosurgery Second Affiliated Hospital, School of Medicine, Zhejiang University Hangzhou China; ^3^ Department of General Practice Second Affiliated Hospital, School of Medicine, Zhejiang University Hangzhou China

**Keywords:** depression, mechanism, migraine, review, therapy

## Abstract

Migraine comorbid with depression is common and is often encountered in clinical practice. The comorbidity may lead to more serious conditions with other symptoms and a longer duration of treatment and it may impose heavy economic and social burdens, directly or indirectly, on patients and their families. Numerous studies have been published on the association of migraine with depression. Numerous literature have showed that the comorbidity may have a common complicated pathogenic mechanism involving biopsychosocial characteristics, including abnormal brain development and shared genetic basis, as well as neurotransmitters, sex hormones and stress. In addition, some studies have identified the multiple, bidirectional relationship between migraine and depressive disorder. We searched the literature for the possible common mechanisms between migraine and depression and classified the research results.

## 1 INTRODUCTION

Headache disorders are common worldwide.^1^ Migraine is a common cerebral disease characterized with a recurrent headache, often accompanied by vomiting, phonophobia and nausea.^2^, ^3^ Migraine is also a paroxysmal headache that affects almost 10% of adults around the world.^1^ The 2015 global disease burden study showed that migraine has become the seventh leading cause of disability in the world.^4^ Studies in the United States, Europe and many other countries show that migraine causes extremely high, direct and indirect economic losses (Table 1).^5^, ^6^, ^7^, ^8^, ^9^

**Table 1:** The mean per‐person annual cost of migraine^7^, ^8^, ^9^

Category	United States	Europe				
		UK	France	Germany	Italy	Spain
Direct costs						
Outpatient care	$424.47	€496.72	€150.75	€317.94	€277.33	€684.48
Procedures	$128.35	€298.95	€82.19	€198.40	€179.98	€324.44
Acute medications	$840.26	€222.81	€295.97	€208.45	€473.74	€207.66
Prophylactics	$279.41					
Other medications	$267.14					
Indirect costs (absenteeism + presenteeism)	$1144.64	€1136 (eight European nations)				

Depression or major depressive disorder is a mood disorder of which the main symptoms are low emotion, lack of interest and pleasure, lack of motivation, decreased self‐esteem, poor sleep and appetite, reluctance to interact with others and reduced productivity.^3^ The lifetime prevalence of depression is approximately 20%, with an annual prevalence of 2%‐5%, and the prevalence is higher in women than men.^3^ Depression is an important cause of social dysfunction. The 2009 report published by WHO predicted that depression will be the primary cause of disease burden by 2030.^10^

As early as 1990, a study enrolling young people in Zürich found a strong connection between migraine and depression.^11^ The link between migraine and depression was also revealed in two Canadian studies involving the general population.^12^, ^13^ Migraineurs also had a higher possibility of depression occurrence than non‐migraineurs.^13^

Migraine often coincides with depression.^14^ People with depression are two to three times more likely to be comorbid with migraine than healthy people.^15^ Furthermore, the presence of depression was a predictor of poor prognosis in patients with a chronic headache.^16^ Migraine and depression often coexist, and their comorbidity may be caused by shared aetiologies. Possible copathogenesis will be discussed below.

## 2 ABNORMAL BRAIN DEVELOPMENT AND BRAIN ACTIVITY

Patients with migraine and depression have a different brain structure compared to the patients suffering from migraine or depression alone. Numerous studies of the structural or functional imaging of migraine have found a difference in the brain and abnormalities in specific cerebral areas.^17^, ^18^, ^19^, ^20^

There are discrepant developmental tracks of the fusiform gyrus, as well as the thalamus, in patients with migraine comorbid with depression. These trajectories are associated with transmission, recognition, control and memory of pain and mood.^17^ The different developmental trajectories suggest that specific mechanisms underlying migraine and depression may contribute to the comorbidity occurrence. A cohort research found that patients with migraine and depression comorbidity have reduced total brain volume, decreased gray and white matter volume, and reduced levels of cerebrospinal fluid compared to patients with only depression, only migraine, or neither.^21^

**Figure 1:**
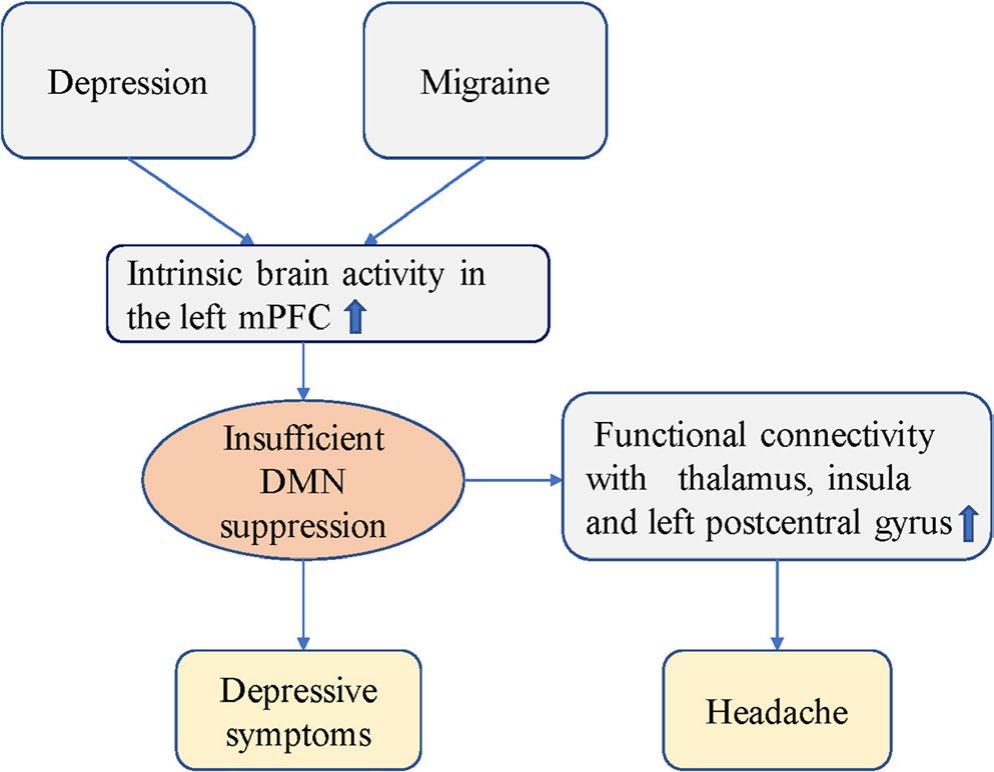
Possible mechanisms of comorbidity through the left mPFC^17^, ^22^, ^23^

A previous study investigated abnormalities in the intrinsic brain activity of patients with the comorbidity using the resting‐state functional magnetic resonance imaging, providing evidence that migraine and depression together influenced the development of the left medial prefrontal cortex (mPFC).^17^ Abnormalities in this brain area can lead to depressive symptoms or migraine symptoms,^17^ the study found increased intrinsic brain activity in the left mPFC in people with depression. The mPFC, as the anterior node of the default mode network (DMN), was highly active at rest but inhibit activity during cognitive and emotional processing, migraineurs also exhibited increased intrinsic brain activity in the left mPFC.^17^ The DMN involves low‐frequency oscillations of about one fluctuation per second. The network is most active when the brain is at rest. When the brain is directed towards a task or goal, the default network deactivates. Areas of the brain included in the default mode network include the medial temporal lobe, the medial prefrontal cortex and the posterior cingulate cortex, as well as the ventral precuneus and parts of the parietal cortex. All of these regions have been associated with some aspect of internal thought. Insufficient DMN suppression was related to increased rumination in depression.^22^ The DMN showed increased functional connectivity with brain areas related to pain during migraine attack, for example, thalamus, insula and left postcentral gyrus.^23^ Based on the above discussion, the possible mechanisms of comorbidity are discussed (Figure 1).

These discoveries may lead to a deeper understanding of the comorbidity and offer a theoretical basis for developing new imaging biomarkers to measure the therapeutic effect. It might be a promising way to explore the relationship between the cerebral cortex thickness and cerebral activity and to understand the copathogenic relationship between cerebral function and depression in patients with migraine.^17^

Other studies have shown that the alterations of regional homogeneity in the caudate nucleus clarified the pathogenesis of migraine, which may be an effective predictor indicating the degree of depression and the progression of migraine without aura.^24^

## 3 SHARED GENETIC BASIS

Many studies enrolling twins found that migraine, as well as depression, shows certain degrees of genetic alterations.^25^, ^26^, ^27^ The familial aggregation of migraine and depression patients suggests that not only genetic but also environmental factors can affect the susceptibility to disease. Considerable genetic contributions can be further identified without environmental considerations. The results provide strong evidence of shared genetic factors.^26^ The heritability of both migraine and depression is approximately 50%,^25^, ^27^, ^28^ with multiple genetic factors.^15^ One research of female twins showed that in more than 1000 pairs of female twins, including both monozygotic and dizygotic, the occurrence of depression was 23% and that of migraine was 20%. The heritability of depression was approximately 58%, whereas that of migraine was approximately 44%. The dual structure equation model determined that 20% of the aberrance in migraine and depression was because of the common genes.^28^ Through the analysis of the largest twin study in Australia to date, the genetic structure of depression and migraine, along with their underlying common genetic ingredients, were assessed. The results suggested that migraine comorbid with depression, either broadly or narrowly defined, can be deciphered by certain common, potentially genetic pathogenesis.^14^

Ligthart et al^29^ showed that migraine and depression are influenced to some extent by the common genes and that the correlation between the hereditary factors affecting both disorders is approximately 0.3 in a two‐variable twin research. Some studies also reported similar consequences.^2^, ^15^, ^28^ Stam et al assessed whether migraine and depression share hereditary factors by choosing more than 2000 families and concluded that there exists a bidirectional association between migraine and depression. Especially, depression and migraine with aura can be partially deciphered by common hereditary factors.^2^ The latest evidence indicated that both diseases share molecular and cellular mechanisms that control both serotonin‐ and glutamine‐neurotransmitter systems. Thus, common hereditary factors may be the basis of migraine and depression comorbidity.^30^, ^31^

The relationship between depression and dopamine deficiency has been demonstrated, showing the hypofunction of the dopaminergic system in patients with migraine,^32^ Stephen et al showed that depression and migraine with aura may be part of syndromes related to an allele mutation of the dopamine receptor gene (Table 2).^33^ As a result, poor dopamine function may lead to depression and migraine comorbidity.^32^

**Table 2:** Candidate gene region, possible mechanism and clinical intervention^33^, ^34^, ^35^, ^36^, ^37^, ^38^, ^39^, ^40^, ^41^, ^42^, ^43^, ^44^, ^45^, ^46^

Candidate gene region	Possible mechanism	Clinical intervention
Dopamine D2 Receptor (DRD2) NcoI Alleles	It affects the expression of dopamine receptors in the presynaptic membrane, which in turn affects the release and reuptake of dopamine in the presynaptic membrane and the binding of the postsynaptic membrane, then affects synaptic transmission and leads to depression and migraine symptoms	The new antidepressant bupropion
Serotonin transporter gene‐linked polymorphic region (SLC6A4)	Depression and migraine symptoms are caused by regulating the serotonin response to stress (affecting serotonin synthesis, transport and binding)	Selective 5‐HT reuptake inhibitors, tricyclic antidepressants and triptans, which are serotonin receptor agonists
Functional polymorphisms of the methylenetetrahydrofolate reductase gene (MTHFR C677T)	Mutations in the MTHFR gene lead to changes in the key enzymes that encode homocysteine and folate metabolism; Elevated homocysteine may lead to endothelial dysfunction, which in turn affects the development of cortical diffusion inhibition leading to migraine, and high homocysteine leads to impaired methylation of the central nervous system leading to depression	Drugs that reduce homocysteine, such as folic acid and B vitamins, may be effective
Cannabinoid receptor 1 (CB1) gene (CNR1)	CNR1 variants are not only associated with neuroticism but also interact with recent life events to predict current depressive symptoms suggests the variants act on the core endophenotypic emotion regulation processes of neuroticism. CNR1 gene is implicated in determining a personality phenotype, may be a vulnerability factor for major depression. Effect of CNR1 on migraine headaches might be related to the alteration of peripheral trigeminovascular activation	An ecb uptake inhibitor (AM404) and a potent CB1 receptor agonist (HU‐210)

In the serotonin transporter gene‐linked polymorphic region, the short ‘s’ allele has inefficient promoter transcription compared with the long ‘l’ allele.^34^ This short ‘s’ allele was associated with the risk of depression.^34^ There is evidence suggesting that a site near the serotonin transporter gene contributes to the genetic susceptibility to migraine.^35^ In addition, there is evidence to show that the ‘s’ allele increases the occurrence of migraine.^36^, ^37^

Functional polymorphisms of the methylenetetrahydrofolate reductase gene (MTHFR C677T) are associated with depressive disorder. Samaan et al showed that MTHFR C677T is associated with the premonitory migraine patients with depression.^38^ The meta‐analysis showed that this genetic variation has a remarkable correlation with premonitory migraine in caucasian people and whole migraine in non‐caucasian people. This interracial influence was demonstrated by the meta‐analysis of 15 studies.

The MTHFR gene encodes key enzymes of homocysteine and folate metabolism.^39^ The MTHFR 677C to T mutation changes a deeply conserved amino acid of this enzyme. Individuals with homozygous mutation exhibited remarkably increased plasma homocysteine levels.^39^ Increased homocysteine levels were observed in patients with migraine with aura.^40^ The increase in homocysteine plasma levels may lead to endothelial dysfunction and then impact the development of cortical diffusion inhibition, which may account for the pathogenesis of migraine with aura.^40^ Many studies have confirmed that hyperhomocysteinemia is associated with depression,^41^, ^47^ and the homocysteine level is positively correlated with the severity of depression. Therefore, we speculate that MTHFR C677T may be associated with the migraine and depression comorbidity.

Juhasz et al demonstrated in an animal experiment that after chronic mild stress the mice deficient of the cannabinoid receptor 1 (CB1) gene (CNR1) showed behaviour of lack of pleasure and helplessness.^42^ In a human study, rimonabant, which is a CB1 antagonist, increased the risk of depressive disorders. The results shown above are the first to show that CNR1 is a risk factor for depressive disorders.^42^ Juhasz et al^43^ showed a remarkable impact of CNR1 on migraine in which the impact is haplotypic but has nothing to do with reported depression. Further study is needed to clarify whether this gene is associated with comorbidities.

Ligthart et al^15^ further studied the genetic overlap between migraine and depression that explored the association of single nucleotide polymorphisms across datasets. Pure migraine and depression are more strongly associated with the substantial decrease in single nucleotide polymorphisms than all migraines and depression, and the observed multiple gene overlap between migraine and depression is because of the characteristics of individual comorbidities.^15^ These results proved the polygenic hypothesis of depression and migraine, and suggested that many single nucleotide polymorphisms do exist, although the risk is small and awaits to be confirmed.

## 4 NEUROTRANSMITTERS AND RECEPTORS

**Figure 2:**
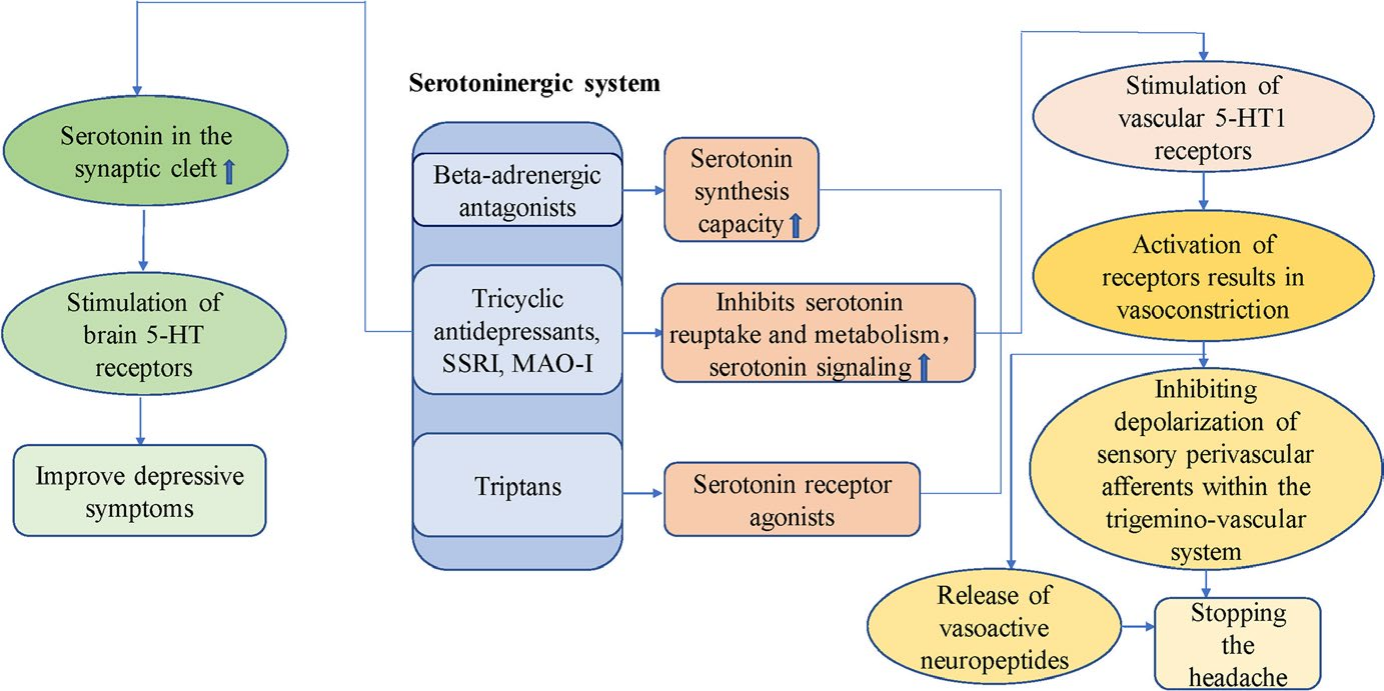
Possible mechanisms by which drugs improve depressive symptoms and migraine symptoms by regulating neurotransmitter and receptor binding^44^, ^45^, ^46^, ^50^, ^51^

Imbalance of the serotonin (5‐HT) neurotransmitters is believed to play an important role in the pathogenic mechanism of major depressive disorder and migraine.^48^ The aetiological theory of depression and migraine focuses on the brain's serotonin function, as both respond to the pharmacological regulators of 5‐HT delivery, such as triptans and selective 5‐HT reuptake inhibitors (SSRIs) (Figure 2).^28^ A possible mechanism is dysfunction of 5‐HT in migraine and mood disorders.^49^

Although migraine and depression are related to the dysfunction of monoamine transmitters, the specific mechanism is still unclear. Although the conclusions of the related research are inconsistent, it is hard to confirm monoamine neurotransmitter as the basis of comorbidity. However, we cannot rule out that there is a common mechanism between depression and migraine involving neurotransmitters.

It is well known that among the current hypotheses of the pathogenesis of depression, the most thoroughly established theory is the monoamine neurotransmitter hypothesis. In this theory, it is believed that a decrease in the monoamine transmitter concentration in the prominent gap is the biological basis of depression. Chugani et al found that the synthesis capacity of 5‐HT increased in migraine patients.^50^ Plasma levels of 5‐HT decreased in migraine attack interphase and increased during migraine attacks.^51^ In one case study of a pair of sisters who had hemiplegic migraine, the systemic 5‐HT levels were low, including levels of 5‐HT metabolite 5‐hydroxyindoleacetic acid (5‐HIAA) in the cerebrospinal fluid and platelet 5‐HT. Interestingly, the use of 5‐hydroxytryptophan improved the symptoms of both sisters.^52^

Some studies found that the increased excretion of 5‐HIAA, which is the main metabolite of 5‐HT, through urine is associated with migraine attacks.^52^, ^53^ Two other studies^54^, ^55^ found a declining tendency in urinary excretion of 5‐HIAA. Bousser et al^55^ presented evidence that urine 5‐HIAA excretion was significantly reduced in 35 adult young female migraineurs between migraine attacks. Milovanovic et al^54^ demonstrated the similar tendency of reduced urinary 5‐HIAA with a small sample (18 migraines).

Therefore, it is possible that 5‐HT may differ between the process and interval of migraine attacks. Consequently, it can be complex and inconsistent to study the common mechanisms of neurotransmitters in migraine and depression.

Previous studies also examined the 5‐HT level in migraine patients and found a low‐5‐HT level.^44^, ^56^ The deficiency or depletion of tryptophan, a precursor to serotonin synthesis, can lead to increased symptoms of depression and migraine.^44^ Moreover, tricyclic antidepressants can further support the impact of 5‐HT on migraine patients via promoting the 5‐HT signalling, thereby lessening the rate of migraine attacks.^45^ Tricyclic antidepressants have been applied to depressive disease patients and were shown to relieve chronic pain. The previous studies found that tricyclic antidepressants can efficiently enhance the concentrations of 5‐HT by preventing the intake of 5‐HT and norepinephrine in the synaptic clearance.^46^ This suggests that there may be a common neurotransmitter mechanism between migraine and depression.

Animal experiments showed that the expression of dopamine and 5‐HT continued to fall in a mouse migraine model.^46^ Repeated use of amitriptyline, which has been a widely used tricyclic antidepressant for half a century, would not increase the level of 5‐HT.^46^ Nevertheless, the levels of 5‐HT and dopamine increased remarkably when amitriptyline was used to cure pain episodes.^46^ Further research is required to identify possible mechanisms by which neurotransmitters interact with both diseases.

However, there are a few fundamental distinctions between migraine and depression in the levels of 5‐HT receptor. The results of positron emission tomography found that the 5HT_1A_ receptors of brain decrease significantly during the depression.^57^ Unlike depression, migraineurs revealed an increase in 5HT_1A_ receptor density.^58^ Furthermore, the first‐line antidepressants, known as SSRIs (selective serotonin reuptake inhibitor), which are used for depression, are not better than placebos for migraine patients.^59^ Instead, the most widely used preventive medicines for migraine, beta blockers, have adverse influences on depression. Therefore, these results do not support the effect of 5‐HT in the occurrence of depression comorbid with migraine. It is contrary to what we have shown before.

## 5 SEX HORMONES

**Figure 3:**
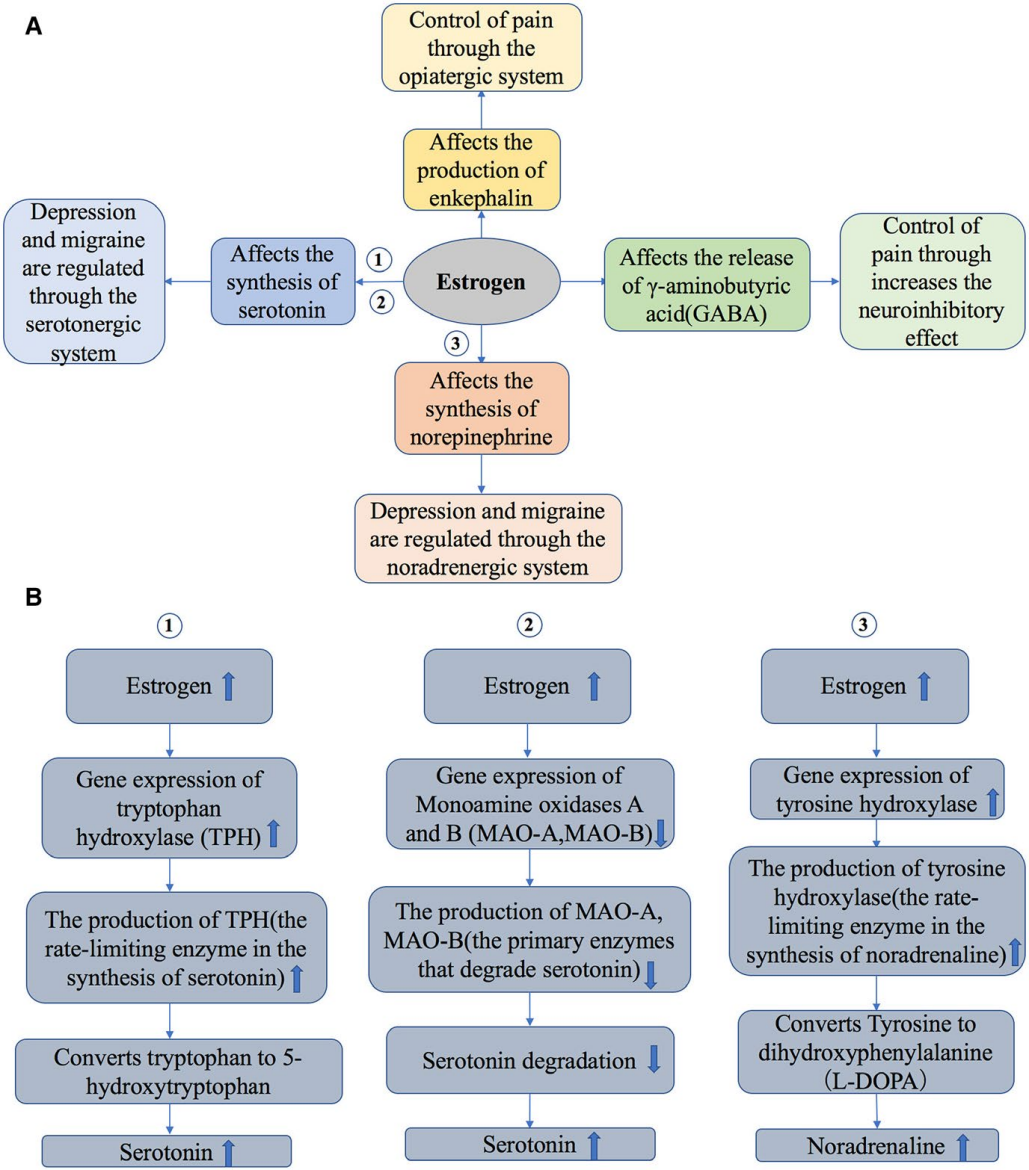
The effects and mechanisms of oestrogen in migraine and depression (A, B)^62^, ^63^

**Figure 4:**
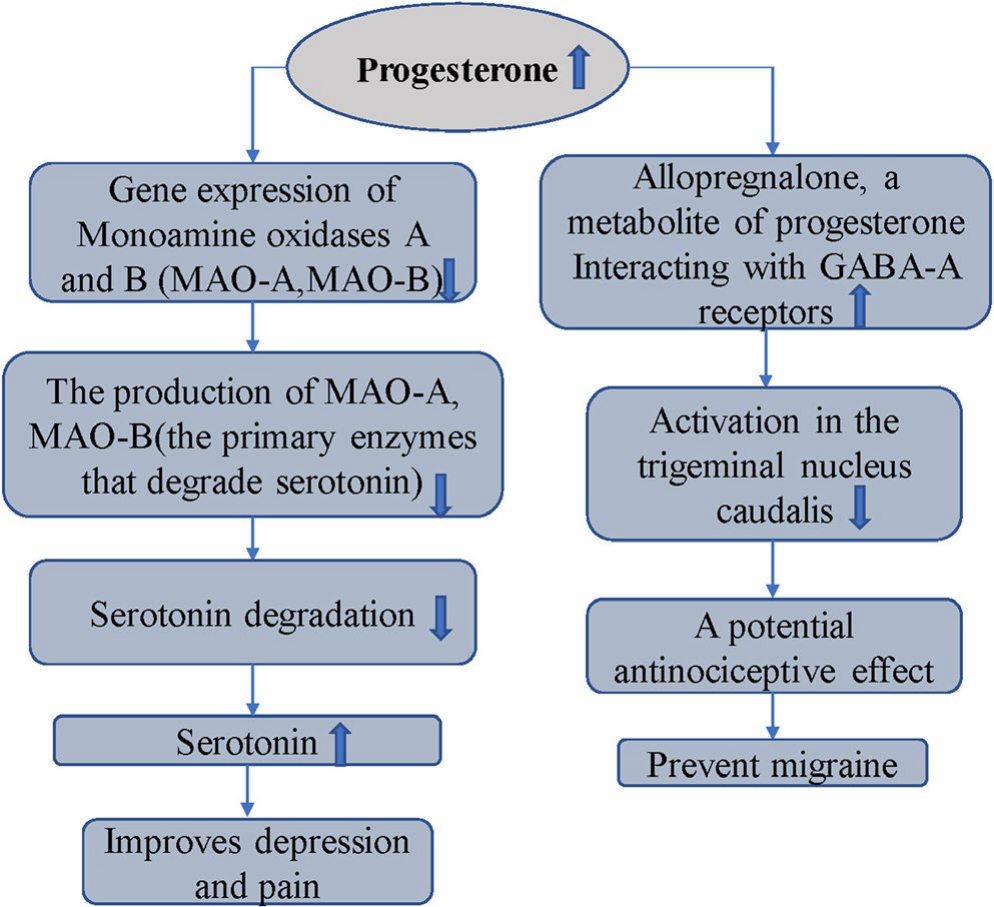
The effects and mechanisms of progesterone in migraine and depression^62^, ^68^, ^69^

The incidence rate of migraine and depression is two to three times higher in females.^10^, ^60^, ^61^, ^62^ It is considered that the alteration in expression of sex hormones exerts an effect in both diseases. Changes in ovarian hormones, particularly oestrogen drop, can lead to downregulation of the 5‐HT energy system and upregulation of the sympathetic system, which lead to migraine comorbid with mood disorders.^62^ The effects and mechanisms of sex hormones in migraine and depression are complex and may involve more than one hormone (Figures 3 and 4).

Many studies have verified that 5‐HT is involved in the physiopathology of migraine and mood diseases (such as major depressive disorders) and that oestrogen could regulate the release of 5‐HT. Furthermore, oestrogen promotes the 5‐HT synthesis by inducing tryptophan hydroxylase, thereby reducing the reuptake of 5‐HT in the nucleus of the dorsal suture.^62^ Moreover, oestrogen could increase the expression of 5‐HT1 receptors, while decreasing the expression of 5‐HT2 receptors. In general, oestrogen exerts agonistic effect on the 5‐HT energy system, which induces a significant connection in the physiopathology between migraine and mood disorders.^63^

Riecher et al^64^ showed that the low oestrogen level triggers depression attacks. The cortical spreading depression (CSD) may be a leading cause of migraine in patients.^56^ Animal studies of female and male mice of type 1 familial hemiplegic migraine have shown that oestrogen elevates sensitivity to cortical diffusion inhibition, which accounts for the progression of migraine with aura.^65^ Although there was a popular notion that migraine can ameliorate after menopause, this theory has yet to be proved. Many migraines in females do not ameliorate, but worsen or even start after menopause.^66^ In addition, the oestrogen replacement therapy may also aggravate the symptoms of migraine.^66^, ^67^ One systemic review concludes that migraine incidence is either steadily sustained or increases after menopause. However, one population‐based study demonstrated that postmenopausal migraines improve.^67^ Oestrogen strengthens a woman's sensitivity to migraine with aura, and oestrogen loss is connected to migraine without aura.^68^ Studies on changes in migraine after menopause are therefore inconsistent.

Progesterone can reduce the expression of monoamine oxidase gene, decrease monoamine oxidase, the major enzyme that can degrade serotonin, increase the level of serotonin in synaptic space and improve depression and migraine.^62^ Animal models of male Hartley guinea pigs with trigeminal neuralgia also showed that the progesterone metabolite can reduce the activity of the trigeminal caudate nucleus. This result may be partially because of its interaction with the GABA receptors, proposing that progesterone has an anti‐injury effect. Therefore, progesterone may prevent migraine (Figure 4).^69^

Research on male mice with familial hemiplegia migraine type 1 proved that susceptibility of CSD was downregulated by testosterone, thereby reducing the occurrence of migraine.^70^ Moreover, testosterone can increase the serotonergic signal,^71^ which suggests that testosterone may lower the incidence of depression.

## 6 SHARED ENVIRONMENTAL FACTORS AND STRESS

To some extent, family clusters of migraine and depression share common environmental factors.^26^ The findings by Schur et al strengthen the feasibility of environmental elements or gene‐environment coactions in the aetiology of migraine and depression.^28^ Gene‐environment coactions, such as those confirmed in depression, may also have an effect on migraine.^34^

A prospective longitudinal study used various stressors (eg, trauma, marriage, economic issues and work stress) to explore the effects of stress on migraine comorbid with depression.^72^ They showed that chronic stress can cause major depression and all types of chronic pain.^72^ Chronic stress was a recognized risk factor for migraine and depression.^73^ The interaction between depression and migraine can largely be explained as stressors that increase the risk of each other.^72^ Stress, as a trigger, can lead to migraine attacks and magnify the frequency and shorten the interval. This research supports the idea that migraine can also be a source of stress, thereby leading to the amplification of the loop, as well as the idea that depression and anxiety also may be the stressors, as these conditions cause the imbalance of homeostasis.^74^ Peterlin et al showed that migraine patients report more stressful life events than those without migraine.^75^

Tietjen et al^76^ recently conducted a clinical research of more than 900 female migraine patients. In this study, women with migraine and depression suffered more childhood abuse than those free of diseases. Previous studies have also shown that childhood abuse can make people more prone to depression and migraine.^76^

Migraine comorbid with depression significantly increases the burden of disease. Therefore, effective stress management strategies for both migraine and depression patients may have a significant impact on preventive and intervening strategies, which may reduce the social costs and burdens of both conditions.^72^

## 7 CAUSAL RELATIONSHIP

The aetiology of migraine is not yet clear, and may be related to certain mental factors such as stress, fatigue, poor mood, excessive or too little sleep. Some people believe that episodes of depression often follow migraine.^11^, ^15^, ^77^ The previous longitudinal studies have confirmed the bidirectional relationship between depression and migraine, which predicts depression and vice versa.^32^, ^72^, ^78^, ^79^, ^80^, ^81^

Twin research has found that among twins with inconsistent anxiety and depression, one of the twins with higher scores for anxiety and depression had a higher frequency of migraine whereas the other did not, although they shared an identical genetic background. In parallel, the risk of anxiety and depression was higher in one of the twins who had migraine.^29^ Another study involving more than 1500 pairs of twins showed a causal relationship between migraine and depression. Without directly involving multiple genes, this causal relationship may account for the comorbidity of depression and migraine.^82^

One possible explanation for the cause‐and‐effect relationship is that severe pain such as migraine may lower the quality of life for patients, thereby leading to depressed symptoms.^15^ Alternatively, migraine can be a manifestation or result of depression. Pain symptoms are frequently found in people with depression; therefore, some researchers believe that pain should be a component of depression.^83^, ^84^ Currently, this assumption lacks solid evidence.

A series of studies conducted by Ligthart et al^15^ explored, via genetic risk scores, the likely cause‐and‐effect relationship between two diseases. There are two likely assumptions as follows: (a) migraines caused by depressive disorder are genetically similar to depressive disorder; and (b) based on the tenet that one disease leads to another, the genes influencing the initial disease must also influence the other disease through the causal chain, and vice versa.^15^, ^82^

Sait et al suggest that depression can cause chronic migraine attacks through central sensitization.^85^ Migraine attacks may be a significant element preventing complete relief of depression.^86^ Migraine is a single predictor of depression.^87^

## 8 CONCLUSION

Data for possible mechanisms of migraine comorbid with depression were insufficient. This paper summarizes the possible common mechanisms of the two diseases and lays a foundation for future research. The possible mechanisms of the migraine and depression comorbidity should be explored in future studies, which could deepen the recognition of the pathophysiology of comorbidities. Identifying common potential genetic factors will help understand the association between migraine and depression and identify new pathways to seek new drug targets.

## CONFLICT OF INTEREST

The authors declare that the research was conducted in the absence of any commercial or financial relationships that could be construed as a potential conflict of interest.

## AUTHOR CONTRIBUTIONS

All authors participated in designing the concept of this manuscript. QZ, AWS and ZYJ reviewed the literature and drafted the article. AWS, HTT and WBL finalized the paper and provided suggestions to improve it.
